# Magnetic Nanoparticle-Based Nano-Packaging and Nano-Freezing in Food Storage Applications

**DOI:** 10.3390/molecules30173453

**Published:** 2025-08-22

**Authors:** Sayan Ganguly, Shlomo Margel

**Affiliations:** 1 Department of Chemistry, University of Waterloo, 200 University Ave West, Waterloo, ON N2L 3G1, Canada; sayanganguly2206@gmail.com; 2 Department of Chemistry, Bar-Ilan Institute for Nanotechnology and Advanced Materials (BINA), Bar-Ilan University, Ramat-Gan 5290002, Israel

**Keywords:** magnetic nanoparticles, food preservation, nano-packaging, nano-freezing

## Abstract

Magnetic nanoparticles (MNPs) have emerged as essential agents in food preservation, tackling significant issues related to shelf life extension, quality maintenance, and safety assurance. This thorough analysis consolidates current developments in MNP-based nano-packaging and nano-freezing technologies, emphasizing their processes, effectiveness, and commercial feasibility. Metallic nanoparticles augment packaging efficacy via antibacterial properties, oxygen absorption, and real-time freshness assessment, while transforming freezing techniques by inhibiting ice crystal development and maintaining cellular integrity. Notwithstanding their potential applications, regulatory uncertainties, toxicity issues, and scalability challenges necessitate collaborative multidisciplinary approaches. We rigorously survey the technological, environmental, and safety aspects of MNP deployment in the food sector and suggest research priorities for sustainable implementation.

## 1. Introduction

The global food system confronts a paradoxical crisis: approximately one-third of all food produced is lost or wasted each year, while over 800 million individuals endure chronic hunger [1]. Post-harvest losses of perishable goods attain alarming rates of 30–40%, chiefly attributable to microbial spoilage, enzymatic degradation, and inadequate preservation technology [2,3,4]. This waste constitutes both a humanitarian crisis and an economic liability of over USD 1 trillion worldwide, accompanied by considerable environmental consequences due to superfluous greenhouse gas emissions and resource exhaustion [5]. Conventional preservation techniques spanning heat processing to chemical additives are increasingly inadequate for modern requirements for minimally processed, additive-free foods with a prolonged shelf life [6]. The seafood industry illustrates these challenges, as traditional freezing leads to harmful ice crystallization, which damages cellular structures, resulting in protein denaturation, drip losses surpassing 15%, and irreversible texture degradation in premium products such as Atlantic salmon (*Salmo salar*) [7]. Recent studies indicate that inadequate freezing–thawing cycles can diminish myofibrillar proteins by 19–37%, significantly undermining nutritional quality and economic value [8]. In this crucial context, magnetic nanoparticles (MNPs) have emerged as transformative instruments, providing unparalleled capacities to tackle preservation difficulties via nanoscale engineering of food matrices and packaging solutions [9]. Magnetic nanoparticles, primarily iron oxides (Fe_3_O_4_, γ-Fe_2_O_3_) and their doped variations, utilize quantum-scale processes to surpass macroscopic preservation constraints [10,11,12]. Their distinctive characteristics, including their superparamagnetism, elevated surface-area-to-volume ratios, and adjustable surface chemistry [13,14,15], provide precise manipulation of biological systems during storage and processing [16,17]. When functionalized with bioactive chemicals or incorporated into polymer matrices, MNPs demonstrate multifunctional properties that surpass traditional preservation methods [18]. The initiation of MNP applications in food systems originates from the research conducted by a group of researchers, who initially established the effectiveness of iron oxide nanoparticles as sorbents for mycotoxin detection in cereals, attaining detection limits ranging from 0.0006 to 1.6337 µg/kg via magnetic micro-dispersive solid-phase extraction methods [19,20,21]. This finding stimulated investigation into wider preservation applications, demonstrating that MNPs might fulfill dual roles: as functional elements in improved packaging and as cryoprotective agents during freezing [22,23]. Zinc-doped ferrite nanoparticles diminished *E. coli* and *S. aureus* populations by over 99% within 24 h in meat packaging systems via reactive oxygen species (ROS) generation, concurrently improving oxygen barrier properties by 68% in polylactic acid films [24]. These discoveries signified a crucial transition from passive confinement to proactive preservation tactics, placing MNPs at the vanguard of food nanotechnology innovation.

The use of MNPs in packaging matrices has revolutionized traditional packaging from simple physical barriers to dynamic, responsive preservation systems [25]. This evolution includes three technical generations, namely enhanced, active, and intelligent packaging systems, with MNPs facilitating functionality in all categories. Active packaging utilizes MNP-facilitated antibacterial properties, gas absorption, and regulated release mechanisms [26]. C18-functionalized multi-walled carbon nanotubes (MWCNTs) in conjunction with Fe_3_O_4_ attained a 92% recovery rate of sulfonamides from milk, significantly exceeding the performance of conventional sorbents [27]. This method illustrates how magnetic separation could optimize extraction procedures while improving sensitivity. Researchers have created Pd-doped MNPs that catalytically convert ethylene, which induces ripening, into acetate and ethanol, thereby delaying climacteric fruit senescence by 40% compared to traditional modified environment packaging [28]. The practical use of these discoveries is demonstrated by products such as Oxy-Guard™ (Fe_3_O_4_-PDMS films) and Nano-Tex^®^ Active (Ag-Fe_3_O_4_ composites), which currently lead niche markets for oxygen-sensitive items like cheeses and fresh vegetables [29]. Intelligent packaging epitomizes MNP innovation, utilizing nanoparticles as transducers in real-time quality monitoring systems [30]. The integration of nanomagnetism with optical sensing principles has facilitated advancements such as anthocyanin-grafted magnetic nanoparticles that exhibit visual color changes upon exposure to amines from rotten fish [31]; this technology has been commercialized as FoodSent sensors [32]. Likewise, superparamagnetic nanoparticles demonstrate irreversible magnetic changes under thermal stress, offering definitive visual proof of cold-chain breaches [33]. Wang et al. [27] explored the quantum mechanical foundations of these phenomena, demonstrating how surface spin disorder in sub-20 nm particles generates field-responsive domains that operate as nanoscale thermometers [34,35]. Recent advancements have progressed from mere indicators to comprehensive diagnostic systems; antibody-conjugated MNPs now facilitate magnetic separation and surface-enhanced Raman scattering (SERS) detection of Salmonella at concentrations as low as 10 CFU/g in poultry, signifying a 100-fold enhancement in sensitivity compared to immunoassays [36]. These solutions convert packaging from static containers into “lab-on-package” platforms that provide real-time safety information, addressing significant deficiencies in supply chain transparency.

Conventional freezing technologies remain plagued by uncontrolled ice nucleation that forms dendritic crystals capable of piercing cell membranes and causing irreversible structural damage [37,38,39,40]. Nano-freezing refers to a process in which the freezing of materials occurs at the nanometer scale, often involving rapid heat transfer and the formation of ice crystals or solid domains confined within nanosized structures. This phenomenon can significantly alter phase transition dynamics, crystal morphology, and material properties compared to conventional freezing [41]. Magnetic nanoparticles overcome this fundamental limitation through four synergistic mechanisms: nucleation modification, ice growth inhibition, magnetic orientation, and targeted nanowarming. A group of researchers revealed that chitosan-coated MNPs (CS@Fe_3_O_4_) serve as heterogeneous nucleation sites, elevating freezing points by 2–3 °C and reducing supercooling by >50% in aquatic products [6]. Another work showed the method of conjugation of herring antifreeze proteins (AFPs) to MNPs, creating “nanoscale ice directors” that adsorb to specific ice crystal planes via hydrogen bonding, restricting Ostwald ripening and producing spherical ice crystals with 90% smaller diameters [42]. Parallel research demonstrated that weak oscillating magnetic fields (5–10 mT) align water molecules into pseudo-crystalline structures that template uniform nucleation; a phenomenon harnessed in commercial systems as shown in the study Effect of Oscillating Magnetic Fields (OMFs) and Pulsed Electric Fields (PEFs) on Supercooling Preservation of Atlantic Salmon (*Salmo salar* L.) Fillets [43]. The most transformative application emerges in magnetic nanowarming, where alternating magnetic fields induce localized heating (42–45 °C) in MNP-embedded tissues, enabling thawing rates 10× faster than conventional methods. Landmark studies on Atlantic salmon filets demonstrated that nano-freezing combined with nano-thawing (NNMT) reduced drip loss by 52%, maintained myofibrillar protein solubility at 87.28% (vs. 64.7% in controls), and suppressed lipid oxidation by 40%, preserving ω-3 polyunsaturated fatty acids [44,45]. Microstructural investigations validated our findings, revealing intact muscle fibers with no protein aggregation. Comparable results were observed in plant-based applications; AFP-MNP coatings diminished ice crystal size by 90% in cherries, maintaining volatile aromatics and textural integrity throughout freeze–thaw cycles. These advancements radically transform cryopreservation from a detrimental necessity to a precise method that preserves cellular architecture.

This comprehensive review distinguishes itself through its integrative examination of MNP applications across the food preservation continuum, from farm-to-fork sensing to cryogenic microstructure control. While previous publications have addressed narrow facets of nanotechnology in food systems, they remain fragmented across disciplinary segments; some focus solely on analytical applications [34], others on biosensing [46,47,48], and several on migration toxicology [49]. This work synthesizes these disparate domains into a unified framework, elucidating how MNPs create synergistic preservation effects when deployed across packaging and freezing applications. The analysis further examines integrated MNP sensors for real-time supply chain monitoring, a synthesis of nanotechnology and digital traceability set to transform food safety. This study contextualizes these advancements within commercial viability and regulatory frameworks, offering not only a technological overview but a guide for responsible innovation. By critically analyzing the most recent studies, we demonstrate that ethically utilized magnetic nanoparticles could diminish global food waste by 15–20% while maintaining nutritional integrity, an essential advancement for sustainable food security amid climate uncertainty and population growth.

## 2. Fundamental Properties and Synthesis of Food-Grade MNPs

Magnetic nanoparticles, particularly iron oxide nanostructures such as magnetite (Fe_3_O_4_) and maghemite (γ-Fe_2_O_3_), have emerged as crucial materials in food preservation due to their distinctive physicochemical properties, biocompatibility, and responsiveness to external magnetic fields [50,51]. The efficacy of MNPs in culinary applications is influenced by their size, morphology, surface charge, crystallinity, and magnetic characteristics, all of which can be precisely adjusted by controlled synthesis and functionalization. At the nanoscale (typically 5–50 nm), MNPs exhibit superparamagnetism [18,52], a phenomenon marked by pronounced magnetic susceptibility when subjected to an external magnetic field, yet demonstrating no residual magnetization after the field is removed, an essential characteristic for recyclable packaging and targeted cryopreservation applications. This feature inhibits particle aggregation during storage while facilitating effective magnetic separation during processing, as evidenced in the formation of reusable MNP-based ethylene scavengers for fruit packing [53]. The elevated surface-area-to-volume ratio of MNPs amplifies their interaction with food matrices, facilitating effective antibacterial activity, gas adsorption, and regulated release of bioactive chemicals.

The synthesis approach significantly affects MNP properties, determining their appropriateness for culinary applications. Co-precipitation is one of the most prevalent procedures, favored for its simplicity and scalability, and entails the alkaline precipitation of Fe^2+^ and Fe^3+^ salts in aqueous solutions. This process generally produces particles ranging from 10 to 30 nm but is afflicted by polydispersity and possible oxide contaminants, requiring post-synthesis purification. To achieve enhanced crystallinity and monodispersity, the thermal breakdown of organometallic precursors, such as iron acetylacetonate, in high-boiling-point solvents like octadecene yields uniform MNPs ranging from 5 to 20 nm; however, the necessity for hazardous organic solvents presents environmental and safety issues [54]. Recent advancements by Abdulwahid et al. have addressed these challenges using solvent-free laser ablation synthesis, producing ultrapure Fe_3_O_4_ nanoparticles with less than 5% size variation, albeit at constrained production sizes [55]. Microemulsion systems, employing water-in-oil droplets as nanoreactors, offer precise size control (5–15 nm) but face challenges in surfactant removal and low yields, obstructing their practical application. Alternatively, hydrothermal/solvothermal synthesis enables the generation of crystalline, defect-free magnetic nanoparticles (20–50 nm) by high-temperature and high-pressure reactions; however, this energy-intensive approach increases both costs and carbon emissions. Innovative green synthesis methods utilizing plant extracts (green tea polyphenols) or microbial agents (generated from *Aspergillus niger*) offer sustainable alternatives that minimize harmful consequences and provide natural stabilizing coverings [56]. MNPs can be biosynthesized using *Aspergillus niger*, where the fungus secretes metabolites that reduce metal salts and facilitate nanoparticle formation. This eco-friendly method offers biocompatibility, controlled particle size, and reduced reliance on toxic chemicals compared to conventional synthesis. Another work was investigated showing that fungal-mediated MNPs had 40% reduced cytotoxicity compared to chemically produced variants, rendering them suitable for food contact applications [57].

Surface functionalization is essential for maintaining colloidal stability, inhibiting oxidation, and customizing magnetic nanoparticles for particular food interactions. Uncoated iron oxide nanoparticles are susceptible to aggregation and fast oxidation to nonmagnetic Fe_2_O_3_ in aqueous settings, undermining performance. To resolve this, polymer coatings including chitosan, polyethylene glycol (PEG), and alginate are often utilized. Chitosan-coated magnetic nanoparticles (CS@Fe_3_O_4_) improve dispersibility and impart inherent antibacterial characteristics, decreasing Listeria monocytogenes levels by 3.5 log CFU/g in poultry packing [58]. Likewise, PEGylation reduces protein corona formation in biological matrices, extending circulation duration in nano-enabled freshness indicators. Silica encapsulation (SiO_2_@Fe_3_O_4_) offers an inert barrier against acid-induced iron leaching [59], which is essential for acidic foods such as citrus juices and fermented products, as uncoated magnetic nanoparticles (MNPs) may raise them above FDA iron migration restrictions (0.5 mg/kg) by as much as 300%. In cryopreservation, biofunctionalization using antifreeze proteins (AFPs), especially type I AFPs derived from winter flounder, facilitates ice recognition functions, allowing magnetic nanoparticles (MNPs) to template hexagonal ice nucleation while preventing recrystallization. A breakthrough was made by demonstrating that AFP-conjugated MNPs decreased ice crystal size in strawberries by 92% relative to traditional freezing methods, thus maintaining cellular integrity and volatile fragrance components [60]. Doping techniques enhance MNP functioning by incorporating secondary metals (Zn, Co, Mn) into the iron oxide lattice. Zinc-doped ferrites (Zn_x_Fe_3−x_O_4_) demonstrate improved antibacterial efficacy by the release of Zn^2+^ ions, resulting in a 99.9% reduction in *E. coli* within 12 h in meat packing films [61]. Manganese doping (Mn_x_Fe_3−x_O_4_) enhances superparamagnetic responsiveness, facilitating ultra-sensitive detection of foodborne pathogens by magnetic relaxation switching assays, achieving limits of detection (LOD) as low as 10 CFU/mL for Salmonella [62]. The magnetic characteristics of doped MNPs are adjustable; cobalt substitution (Co_x_Fe_3−x_O_4_) enhances coercivity, rendering the particles appropriate for high-density data storage in smart packaging labels that document time–temperature histories. Nonetheless, doping presents regulatory challenges, as transition metals may leach into food. Comprehensive leaching studies conducted by the European Food Safety Authority (EFSA) demonstrated that silica-coated Zn-doped MNPs released less than 0.1 mg/kg of Zn^2+^ in fatty foods, much below the 5 mg/kg safety limit [63]. Scalability and regulatory compliance continue to pose significant obstacles in the commercialization of MNPs. Inconsistencies across batches in co-precipitation—caused by minor pH and temperature fluctuations—can modify particle size distributions by as much as 35%, affecting performance repeatability. Continuous-flow microreactor systems have emerged as a solution, yielding MNPs with less than 8% size variability in kilogram-scale outputs, as confirmed by BASF’s pilot production trials. The regulatory frameworks are changing; the FDA designates iron oxide MNPs as GRAS (21 CFR 73.200) under certain conditions, whereas the EU’s Novel Food Regulation (2015/2283) requires individual safety evaluations for surface-modified variations. There is an urgent necessity for harmonized norms, especially with doped and composite MNPs, as existing laws are insufficiently aligned with technological progress [64]. The methodical design of food-grade magnetic nanoparticles relies on a careful equilibrium among magnetic efficacy, colloidal stability, and biocompatibility, accomplished by customized synthesis and surface modification. Future directions encompass AI-driven synthesis optimization to minimize defects, biodegradable coatings such as polylactic acid (PLA) to improve sustainability, and modular functionalization for multifunctional applications, including concurrent pathogen detection and antioxidant release. As the discipline advances, multidisciplinary collaboration among chemists, food scientists, and regulators will be crucial for converting laboratory ideas into safe, scalable, and socially accepted food preservation solutions.

## 3. Nano-Packaging as Active and Intelligent Systems

**Figure 1:**
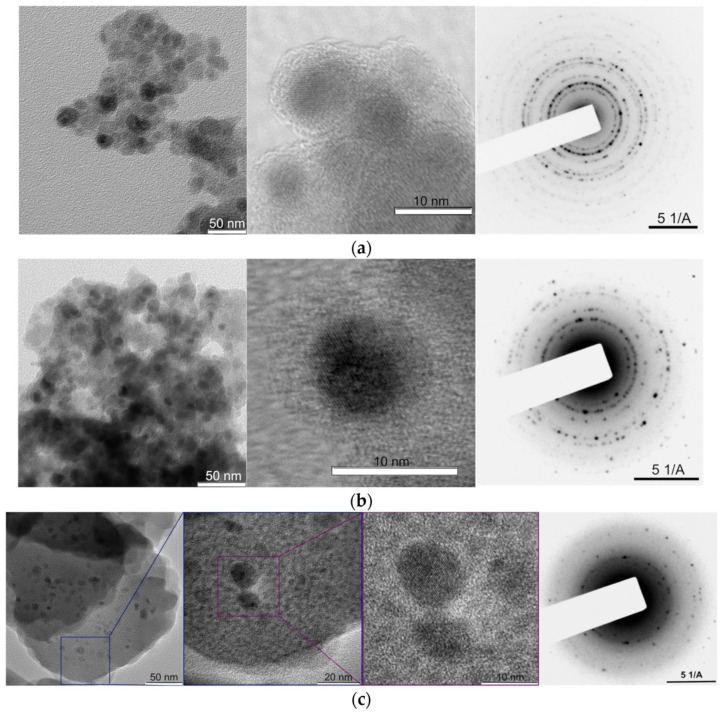
The electron diffraction patterns and transmission electron micrographs of (**a**) MNP 1, (**b**) MNP 2, and (**c**) the substance that was produced by heating MNP 1 at 180 degrees Celsius for forty-five hours [71].

The incorporation of MNPs into food packaging has revolutionized conventional materials by introducing active and intelligent functionalities, transforming passive barriers into dynamic systems that interact with food and the environment [65]. Active packaging employs MNPs to extend shelf life by inhibiting microbial growth, scavenging harmful gases, or releasing preservatives, while intelligent packaging uses MNPs as sensing elements to monitor food quality in real time [66]. These advancements address critical challenges in food preservation, including microbial spoilage, oxidation, and supply chain monitoring, offering solutions that are both efficient and sustainable. MNPs enhance active packaging through their innate antimicrobial properties and ability to be functionalized with bioactive compounds [67]. Iron oxide nanoparticles (Fe_3_O_4_) doped with silver (Ag) or zinc oxide (ZnO) have potent bactericidal properties due to their release of metal ions (Ag^+^, Zn^2+^) that compromise microbial cell membranes and produce reactive oxygen species (ROS). Research indicates that Ag-Fe_3_O_4_ nanocomposites diminish *E. coli* and *S. aureus* populations by over 99% within 24 h in meat packaging, surpassing conventional antimicrobial films. Chitosan-coated magnetic nanoparticles infused with essential oils, such as thyme or oregano oil, facilitate the regulated release of natural antimicrobials, prolonging the shelf life of fresh produce by as much as 50% relative to traditional packaging methods [68]. Beyond antimicrobial activity, MNPs play a crucial role in gas scavenging, particularly in controlling oxygen and ethylene levels. Oxygen-sensitive foods, such as meats and dairy products, benefit from MNP-embedded films that catalytically decompose O_2_. For instance, palladium-doped MNPs (Pd-Fe_3_O_4_) integrated into polylactic acid (PLA) films reduce oxygen permeability by 68%, significantly slowing lipid oxidation and rancidity [69]. In fresh produce packaging, MNPs functionalized with potassium permanganate (KMnO_4_) effectively scavenge ethylene, delaying fruit ripening by up to 40% compared to standard modified atmosphere packaging [70]. Magnetic nanocomposite materials comprising Fe_3_O_4_ nanoparticles coated with iron and silica glycerolates (MNP@Fe(III)Glyc and MNP@Fe(III)/SiGlyc) were synthesized. The generated nanocomposites were analyzed by TEM, XRD, TGA, VMS, Mössbauer, and IR spectroscopy. The quantities of iron and silica glycerolates in the nanocomposites were determined using Mössbauer spectroscopy, ICP AES, and C,H-elemental analysis. The distribution of Fe in the shell and core for MNP@Fe(III)Glyc and MNP@Fe(III)/SiGlyc was 27:73 and 32:68, respectively (Figure 1). The produced nanocomposites exhibited elevated specific magnetization values and a pronounced magnetic response to the alternating magnetic field. The hydrolysis of shells composed of Fe(III)Glyc and Fe(III)/SiGlyc in aqueous environments was examined [71].

**Figure 2:**
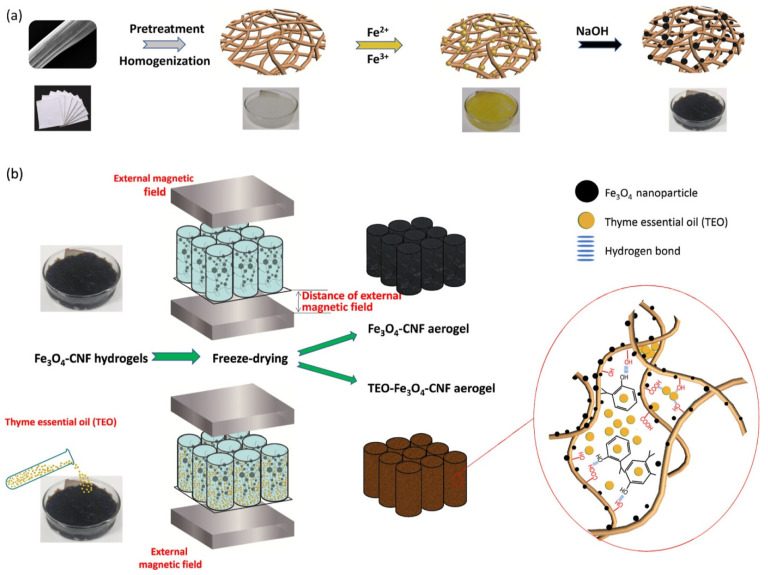
Schematic illustration of the formation of a (**a**) Fe_3_O_4_–CNF hydrogel, (**b**) Fe_3_O_4_–CNF aerogel, and TEO–Fe_3_O_4_–CNF aerogel [76].

Essential oils (EOs) are preferred in the research of eco-friendly active food packaging due to their superior antibacterial and antioxidant properties [72,73]. Nevertheless, owing to their offensive odor and significant volatility, it is imperative to regulate their concentration over time post-release to prevent excessive odorant contamination of the intended products and preserve their antibacterial efficacy [74,75]. Another work presented a simple and eco-friendly method utilizing cellulose nanofibers (CNFs), iron oxide (Fe_3_O_4_), and thyme essential oil (TEO) to produce TEO/Fe_3_O_4_/CNF aerogels with a micro/nanoporous structure for the controlled release of volatile TEO, aimed at prolonging food shelf life and maintaining their organoleptic properties [76]. The chemical deposition approach was employed in situ to mineralize magnetic nanoparticles (NPs) with significant antibacterial efficacy on the surface of CNF (Figure 2). An external magnetic field was utilized to facilitate fiber movement and regulate the aerogel pore structure throughout the freeze-drying process, significantly influencing the storage and release of TEO. The concurrent synergistic interaction between the TEO and Fe_3_O_4_ nanoparticles, with contact bacteriostatic activity, imparted the aerogel with exceptional antibacterial efficacy.

**Figure 3:**
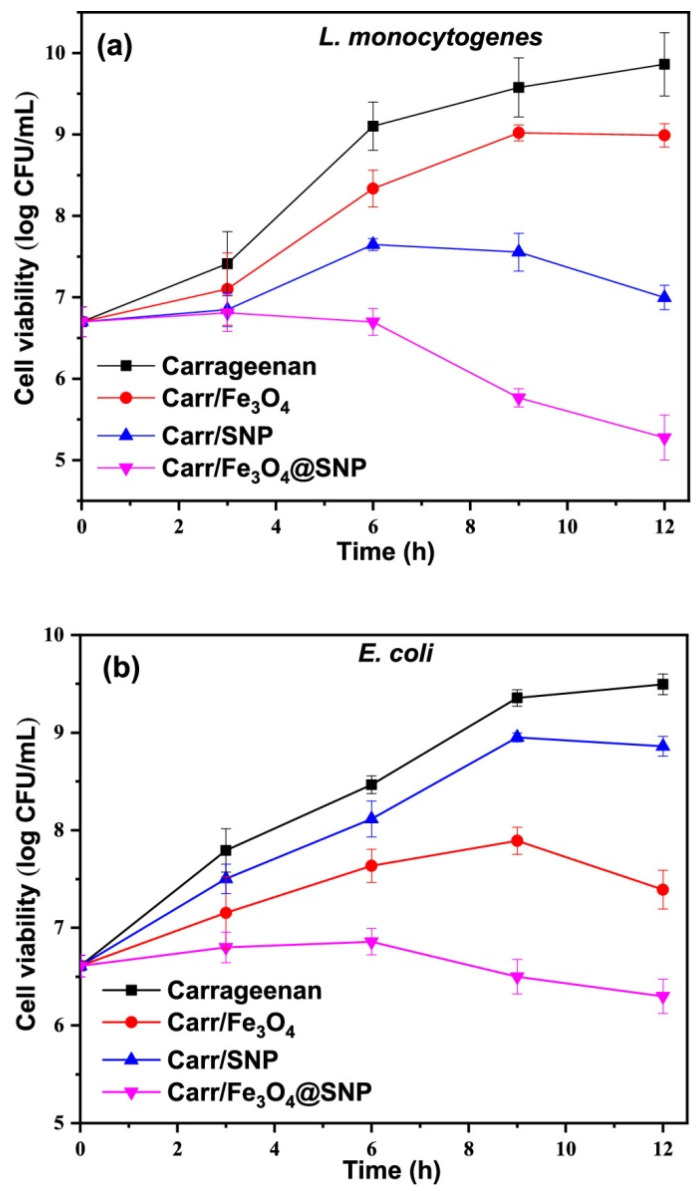
Antibacterial activity of carrageenan-based films against *L. monocytogenes* and *E. coli* [79]. Here, SNP stands for silica nanoparticle.

The post-harvest loss of agricultural products poses a significant issue for farmers, distributors, buyers, and consumers, as it substantially affects the economic aspects of large-scale agriculture. This degradation mostly arises from the attack of numerous microbes and exposure to many atmospheric chemicals and gases, such as oxygen, carbon dioxide, and water, leading to a decline in visual appeal, nutritional value, and quality, as well as a decrease in product shelf life [77]. Nanotechnology has led to creative solutions to this issue through the application of nanomaterials, including nanoparticles, nanocomposites, and nano-films, in coating, packaging, and diagnostics [78]. Sulfur-coated Fe_3_O_4_ hybrid nanoparticles (Fe_3_O_4_@SNP) featuring a core–shell architecture were produced. Functional nanocomposite films of carrageenan were synthesized by incorporating Fe_3_O_4_, SNP, and Fe_3_O_4_@SNP nanoparticles. Their properties, such as mechanical strength, UV–vis barrier, water vapor permeability (WVP), surface morphology, thermal stability, water contact angle (WCA), surface color, and antibacterial activity, were analyzed [79]. Figure 3 illustrates the outcomes of the antibacterial efficacy of carrageenan-based films against *E. coli* and *L. monocytogenes*, as assessed by the total viable colony count method. The carrageenan films infused with Fe_3_O_4_ and silica nanoparticles (SNP) exhibited antibacterial activity against *E. coli* and *L. monocytogenes*, demonstrating a degree of reduction in growth rate. The antibacterial efficacy of the composite films varies according to the bacterial type. The SNP-infused film exhibited superior antibacterial efficacy against the Gram-positive bacterium (*L. monocytogenes*) compared to the Gram-negative bacterium (*E. coli*); conversely, the Fe_3_O_4_-infused film showed enhanced activity against the Gram-negative bacteria relative to the Gram-positive bacteria. The antibacterial properties of sulfur are well established, demonstrating significant bactericidal effects against Gram-positive bacteria.

**Figure 4:**
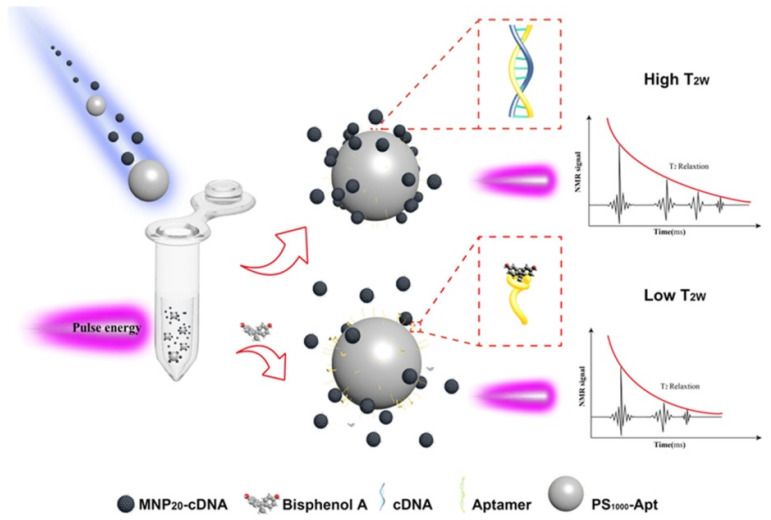
Schematic illustration of the PS-MRS (polystyrene–magnetic resonance sensor) designed for the detection of bisphenol A (BPA). This Figure depicts the structural configuration of the sensor, highlighting the functional polystyrene matrix, the embedded magnetic nanoparticles that enhance sensitivity, and the recognition sites specific to BPA molecules [80].

Bisphenol A (BPA) is a harmful endocrine disruptor. Thus, there exists a considerable demand for rapid and precise methodologies for the quantification of BPA. Huang et al. developed a technique for enhancing relaxation signals using a magnetic relaxation switch (MRS) biosensor, which relies on the self-assembly of polystyrene microspheres and magnetic nanoparticles to identify bisphenol A (BPA) in food packaging materials and water samples [80]. Aptamer-functionalized polystyrene microspheres (PS1000–Apt) and complementary DNA-functionalized magnetic nanoparticles (MNP20–cDNA) underwent self-assembly to create a hybrid chain (Figure 4). The MNP20–cDNA could be sequestered by the enhanced size and augmented aptamer loading of PS1000–Apt, subsequently transitioning from dispersion to aggregation. The aptamers demonstrated great affinity and selectivity for BPA, resulting in the formation of stable “PS1000–Apt–BPA” complexes and a reduction in MNP20–cDNA aggregation. A modified relaxation signal was obtained. The experimental results confirmed that the suggested biosensor facilitated accurate detection of BPA. Under optimal detection conditions, a detection range of 0.1–100 ng/mL and a lower limit of detection (LOD) of 0.06 ng/mL were attained, in contrast to the conventional MRS LOD of 0.36 ng/mL. The method was successfully employed for the identification of BPA in water and polycarbonate bottle samples. These PC bottles are appropriate for holding beverages, juices, and mineral water.

**Figure 5:**
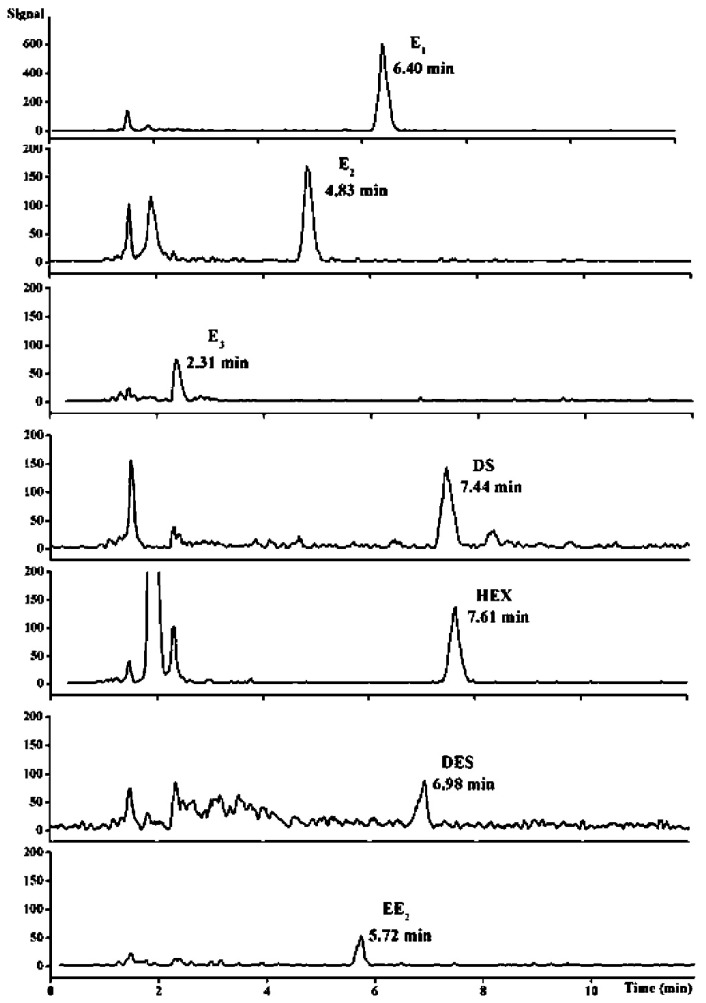
LC–MS/MS chromatograms of seven estrogens extracted by MNPs/PPy from the spiked milk sample containing 0.5 ng/mL of each estrogen [81].

A nanocomposite of polypyrrole-coated magnetite nanoparticles (designated as MNPs/PPy) was synthesized and utilized as a magnetic solid-phase extraction (MSPE) sorbent for the extraction of estrogens from milk samples. The polypyrrole coating, distinguished by a robust π-conjugated structure and hydrophobic characteristics, allowed MNPs/PPy to demonstrate remarkable effectiveness in estrogen extraction. Estrogens can be readily isolated from milk samples via MNPs/PPy, eliminating the necessity for protein precipitation. Moreover, the extraction may be finalized within 3 min [81]. A swift, straightforward, and efficient technique for analyzing estrogens in milk samples was developed by integrating MNPs/PPy-based MSPE with liquid chromatography–tandem mass spectrometry (LC–MS/MS). The detection limits for the estrogens examined ranged from 5.1 to 66.7 ng/L (Figure 5). The recoveries of the estrogens (concentration range of 0.5–20 ng/mL) from the milk samples were between 83.4% and 108.5%, with relative standard deviations from 4.2% to 15.4%.

## 4. Nano-Freezing Technologies

Conventional freezing methods, while widely used for food preservation, are impeded by a major disadvantage: the formation of large, irregular ice crystals that damage cellular structures, leading to texture degradation, nutrient loss, and considerable drip loss upon thawing [82]. The structural damage is especially significant in high-value items like shellfish, berries, and leafy greens, where cellular integrity is directly linked to quality. Nano-freezing technologies, utilizing the distinctive characteristics of magnetic nanoparticles (MNPs), have arisen as a revolutionary solution to these issues by facilitating regulated ice nucleation, growth suppression, and ultra-rapid nanowarming [83]. These developments are based on the superparamagnetic properties of iron oxide nanoparticles (Fe_3_O_4_, γ-Fe_2_O_3_), enabling precise control of ice crystallization dynamics under external magnetic fields, while ensuring food safety and scalability [84]. The core premise entails MNPs functioning as nanoscale ice regulators, either by serving as nucleation templates to reduce supercooling or by adhering to ice crystal surfaces to inhibit recrystallization, yielding smaller, more uniform ice crystals that maintain their microstructure.

**Figure 6:**
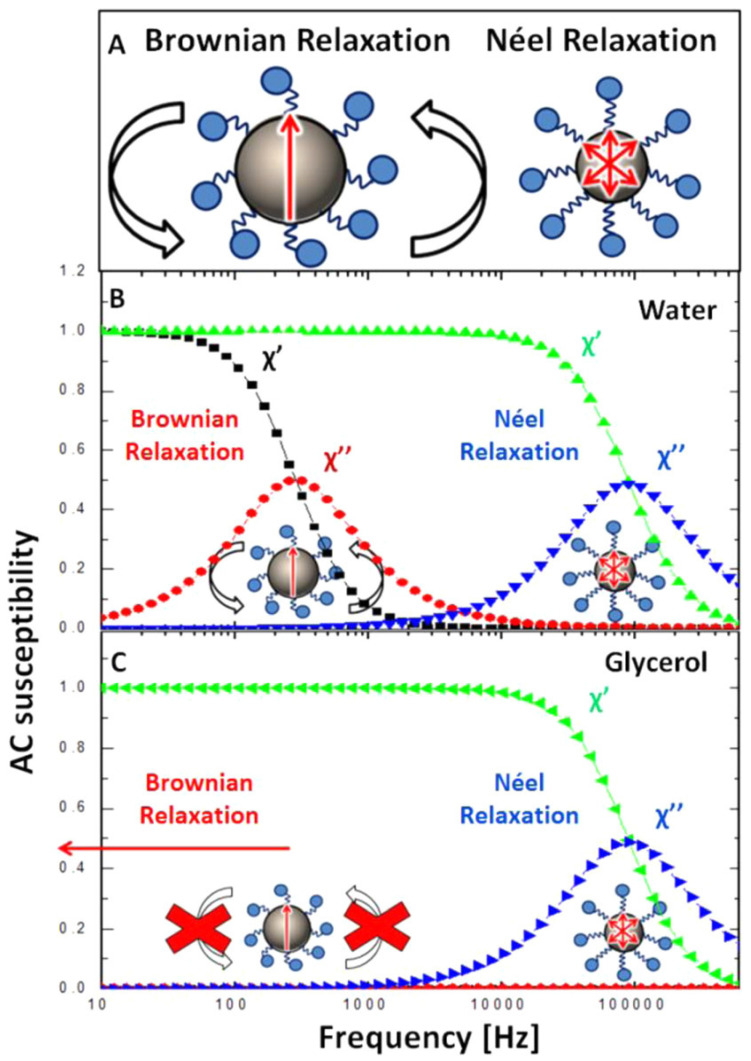
Schematic depiction of the Brownian and Néel relaxation mechanisms of nanoparticles (**A**) alongside their respective AC susceptibility curves in water (**B**) and glycerol (**C**), illustrating low- and high-viscosity dispersions, respectively. The in-phase and out-of-phase components of the complex AC susceptibility are denoted as χ′ and χ″, respectively [85].

Magnetization relaxation mechanisms profoundly influence the behavior of magnetic nanoparticles in high-frequency fields, particularly in applications such as magnetic hyperthermia. The existing procedure depends on particle mobility, which is subsequently affected by the environment [85,86,87]. A study utilized AC susceptometry to observe the in situ magnetic response of model systems consisting of blocked and superparamagnetic nanoparticles after their cellular absorption and subsequent release using freeze–thaw lysis [88]. The AC susceptibility signal from internalized particles in living cells demonstrated only Néel relaxation, consistent with findings from immobilized nanoparticle suspensions [89]. Nonetheless, Brownian relaxation was restored after cell lysis, indicating that the immobilization effect was reversible and that the nanoparticles’ integrity was maintained within the cells (Figure 6). The results indicate that cellular internalization can inhibit Brownian relaxation, which has important implications for the design of appropriate nanoparticles for intracellular hyperthermia applications [85].

**Figure 7:**
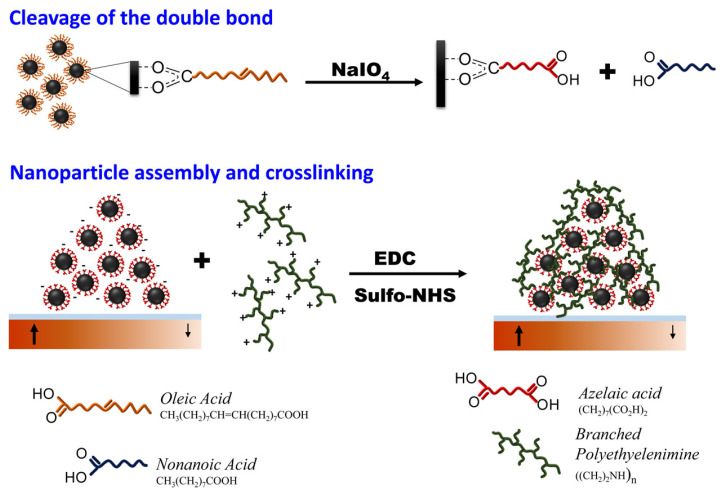
Schematic representation of the cross-linking process in iron oxide nanoparticles, initiated by the creation of oleic acid following thermal breakdown of the particles. The breaking of the double bond in oleic acid generates a terminal carboxylic group that can facilitate the cross-linking of particles with a polyamine like PEI through EDC chemistry [90].

Velez et al. delineated a multifaceted technique for the fabrication of magnetic microstructures including intricate two-dimensional geometric configurations, utilizing magnetically integrated iron oxide (Fe_3_O_4_) and cobalt ferrite (CoFe_2_O_4_) nanoparticles. Magnetic pole patterns are encoded in magnetizable media, upon which magnetic nanoparticles are organized from a colloidal suspension into specific configurations by structured magnetic field gradients. The kinetics of the assembly process are analyzed by assessing microstructural characteristics (e.g., line width and height) in relation to time, particle type, and volume fraction (Figure 7). Upon assembly, the iron oxide particles undergo in situ cross-linking and are subsequently liberated by the dissolution of a sacrificial layer [90].

**Figure 8:**
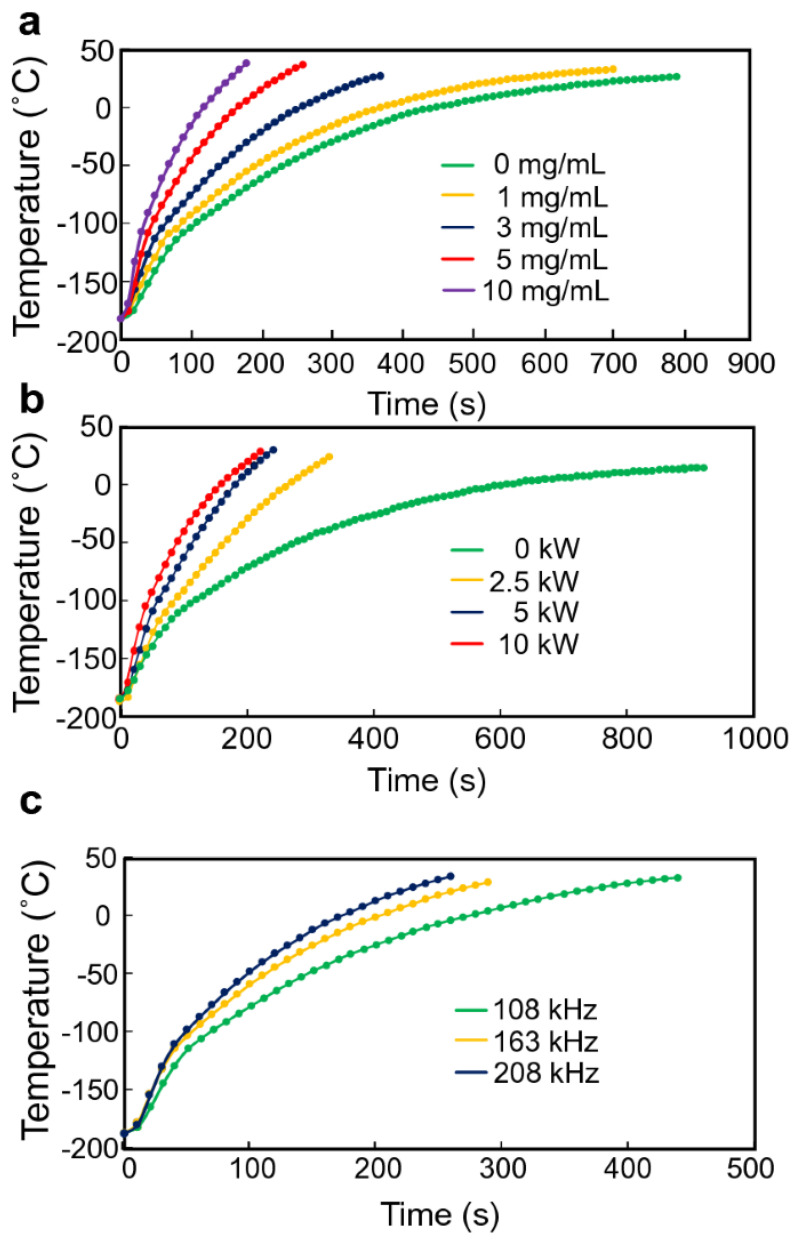
(**a**) Irradiated temperature profiles during alternating magnetic field. Magnetite nanoparticles were inductively heated to nanowarm vitrified StemCell Keep. Representative vial center temperatures are shown. Nanowarming power output, 10 kW; magnetite concentration, 0 mg/mL (green), 1 mg/mL (yellow), 3 mg/mL (blue), 5 mg/mL (red), and 10 mg/mL (purple); frequency, 208 kHz. (**b**) Nanowarming power output, 0 kW (green), 2.5 kW (yellow), 5 kW (blue), and 10 kW (red); magnetite concentration, 5 mg/mL; frequency, 208 kHz (**c**). Nanowarming power output, 10 kW; magnetite concentration, 5 mg/mL; frequency, 108, 163, and 208 kHz [92].

Semen was cryopreserved using either glycerol-based or sucrose-based extenders. Freeze–thawed straws from five donkeys (three ejaculates per donkey) were incubated with lectin–MNPs (2 mg/mL) and subsequently subjected to an external magnet, allowing for the collection of non-bound sperm as nanopurified sperm. The sperm was evaluated post-thawing (control) and following nanopurification for motility, plasma membrane integrity, acrosome integrity, morphology, DNA fragmentation, and concentration. The statistical studies were broadened to investigate the correlation between the initial quality of the freeze–thawed semen samples and the effects of nanopurification following thawing. The obtained MNPs demonstrated biocompatibility with sperm and significantly improved progressive motility (*p* < 0.05) in the glycerol nanopurified group (43.08 ± 3.52%) relative to the control group (33.70 ± 2.64%) [91]. Acrosome-damaged sperm was significantly decreased (*p* < 0.05) in both nanopurified groups (19.92 ± 2.69 for G and 21.57 ± 2.77 for S) compared to the control (36.07 ± 3.82 for G and 35.35 ± 3.88 for S). No substantial alterations in sperm morphology and membrane integrity were observed following nanopurification. The mean sperm recovery following nanopurification was 80.1%. The sperm quality index was markedly elevated (*p* < 0.001) in the nanopurified groups, irrespective of the baseline quality of the freeze–thawed semen samples. Ito et al. presented a scalable nanowarming technique for hiPSC cryopreservation that employs inductive heating of magnetic nanoparticles within an alternating magnetic field. The conventional method of heating with a water bath at 37 °C caused a decline in cell viability due to devitrification from the incremental warming of samples with substantial volumes (≥20 mL). Nanowarming exhibited reliable and rapid rewarming of the vitrified samples, improving the viability of hiPSCs in the 20 mL system. Furthermore, hiPSC aggregates produced by a bioreactor approach were successfully cryopreserved using nanowarming technology, alongside individual cells (Figure 8). These findings indicate that nanowarming is a viable technique for the cryopreservation of large quantities of hiPSCs [92].

**Figure 9:**
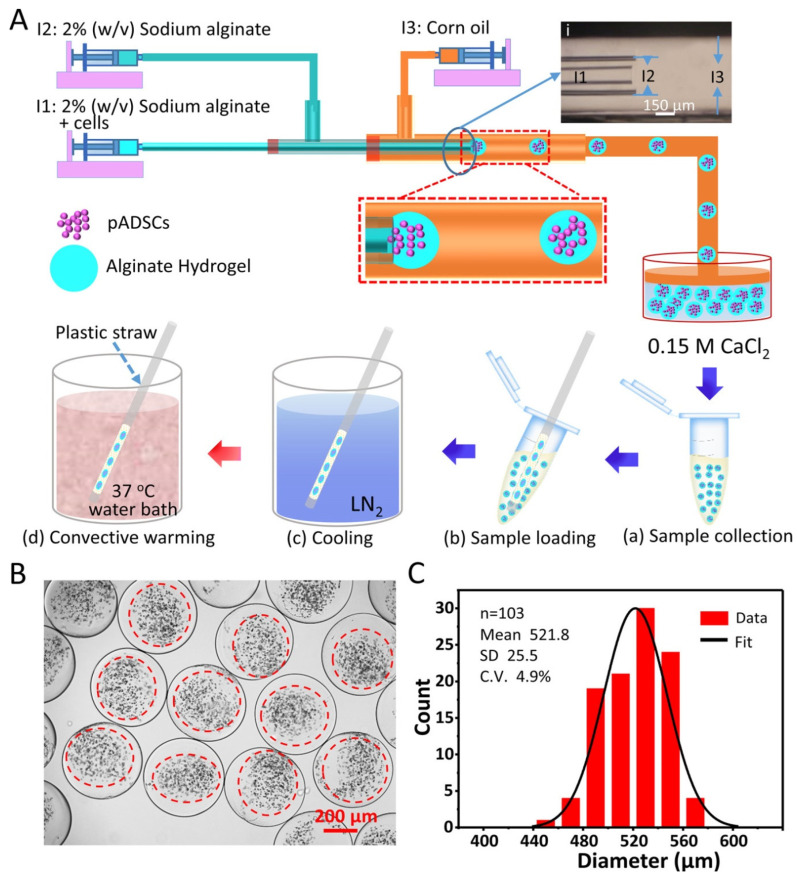
Schematic illustration of generating cell–alginate hydrogel biocomposites by using a tube-in-tube capillary microfluidic device and the procedure of vitrification with warming at 37 °C in a water bath. (**A**) Overview of the device, the procedure for generating microcapsules, and the procedure for vitrification with warming at 37 °C in a water bath. (**i**) Micrograph showing the configuration of the tube-in-tube capillary device. The sketches in panels a–d illustrate the process of vitrification with warming at 37 °C in a water bath. (**B**,**C**) Typical differential interference contrast (DIC) image and diameter distribution of the microcapsules generated using the tube-in-tube capillary microfluidic device in this study. C.V.: Coefficient of variation. Red dotted circles are showing the localization [105].

Nanosized rifampicin (RIF) has been synthesized to enhance solubility in aqueous solutions, resulting in significant improvements in bioavailability. This is facilitated by a novel, nontoxic, and biodegradable delivery system utilizing magnetic iron oxide nanoparticles (MIONs) cross-linked with polyethylene glycol hybrid chitosan (mCS-PEG) gel beads. The functionalization of nano RIF and mCS-PEG gel beads was examined utilizing diverse spectroscopic and microscopy methodologies. The dimensions of the synthesized nano RIF were determined to be 70.20  ±  3.50 nm. The incorporation of PEG enhanced the mechanical stability and swelling ratio of the magnetic gel beads, achieving a maximum swelling ratio of 38.67  ±  0.29 g/g. These magnetic gel beads possessed dual responsive properties for nano drug delivery applications, specifically in terms of pH and magnetic fields [52,93,94]. As anticipated, the magnetic gel beads exhibited a superior nano drug release capability in an acidic medium (pH  =  5.0), with a maximum efficiency of 71.00  ±  0.87% [95]. Yan et al. introduced the term “nanocryosurgery” to describe an innovative surgical approach that integrates the principles of cryosurgery and nanotechnology for effective tumor therapy [96]. Simulations were conducted on the integrated phase change bioheat transfer concerns at the single cell level and its adjacent tissues to elucidate the disparity in transient temperature responses between traditional cryosurgery and nanocryosurgery [97]. Theoretical interpretations and current experimental data indicate that deliberately introducing nanoparticles with high thermal conductivity into target tissues can substantially reduce the final temperature, enhance the maximum freezing rate, and increase the ice volume compared to conditions without nanoparticles [98,99,100]. The incorporation of nanoparticle-enhanced freezing may render conventional cryosurgery more versatile by allowing artificial manipulation of the size, shape, appearance, and orientation of ice ball production [96]. MNPs inhibit ice nucleation and growth during the processes of freezing and thawing. This study examined the impact of MNP-assisted cryogenic freezing combined with MNP-enhanced microwave thawing (NNMT) on the thermodynamic properties and qualitative alterations in salmon filets. The findings indicate that NNMT elevates Tg (glass transition temperature) and T_max_ (transition temperature), hence enhancing the storage stability of salmon filets [101]. The MNP-assisted freezing and thawing treatment, particularly NNMT therapy, markedly enhanced the water retention capacity, texture, color, and other quality attributes of salmon filets. The NNMT therapy exhibited the lowest degrees of lipid and protein oxidation, while its myofibrillar protein solubility reached its maximum level at 87.28%. Superparamagnetic iron oxide nanoparticles exhibiting pronounced nonlinear magnetic properties are appealing for biomedical applications such as magnetic particle imaging and magnetic fluid hyperthermia [17,102]. These particles have intriguing magnetic characteristics in alternating magnetic fields, and we present studies that reveal variations in the magnetization dynamics of certain particles in both frozen and molten states. This effect transcends the minor temperature differential (Δ*T*~20 °C), and we demonstrate that the dynamics comprise a combination of Brownian alignment of the particles and Néel rotation of their moments in liquid particle suspensions [103]. The phenomena can be represented using a stochastic differential equation framework by assuming log-normal distributions and partial Brownian alignment of an effective anisotropy axis [103]. Janus magnetic nanoparticles (approximately 20 nm) were synthesized by grafting either polystyrene sodium sulfonate (PSSNa) or polydimethylamino ethylmethacrylate (PDMAEMA) onto the exposed surfaces of negatively charged poly(acrylic acid) (PAA)-coated magnetite nanoparticles adsorbed onto positively charged silica beads. Individually dispersed Janus nanoparticles were obtained by repulsion from the beads following the reversal of the silica surface charge due to an elevation in the solution pH. At low pH levels, the regulated aggregation of Janus nanoparticles led to the formation of stable clusters around 2–4 times the original particle size. Cluster formation was reversed, and individually dispersed nanoparticles were restored by increasing the pH to elevated levels [104]. Microencapsulated stem cells in hydrogel, as stem cell–hydrogel constructions, offer extensive applications in the emerging field of cell-based medicine. Given their limited shelf life at room temperature, long-term storage or banking of the structures is crucial for the immediate availability required for extensive applications. Low-cryoprotectant (CPA) vitrification has garnered significant interest recently as a high-efficiency, user-friendly, low-toxicity, and cost-effective approach for the long-term storage of structures. Nevertheless, we observed that numerous cells within the stem cell–alginate constructions (about 500 μm in diameter) failed to adhere to the substrate following low-CPA vitrification using around 2 M penetrating CPAs. To resolve this issue, nanowarming has been implemented by magnetic induction heating (MIH) of Fe_3_O_4_ nanoparticles to reduce recrystallization and devitrification during the warming phase of the low-CPA vitrification process (Figure 9). The findings of this study demonstrate that high-quality stem cell–alginate hydrogel constructs, characterized by an intact microstructure, elevated immediate cell survival (>80%), and significantly enhanced attachment efficiency (approximately threefold, 68% compared to 24%), can be achieved following cryopreservation using nanowarming [105].

**Figure 10:**
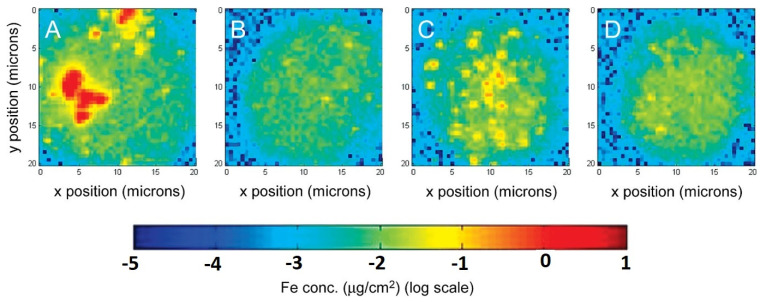
Representative false-color XRF microscopy images showing iron content for a KB cell from each of four populations: (**A**) KB-FAR+ with 50 nM DC-SPIONs, (**B**) KB-FAR+ with 50 nM DC-SPIONs + free FA, (**C**) KB-FAR with 50 nM DC-SPIONs, and (**D**) untreated control. All incubations were for 1 h. The qualitative feature of localized points of high iron concentration in (**A**) is obvious; these pockets overwhelm the signal from the cell’s endogenous iron background [107].

The iTRAQ-based quantitative proteomic method was utilized to clarify the effect of magnetic nanoparticles combined with microwave thawing (MNPMT) on the quality of largemouth bass filets. A total of 47 proteins were identified as differentially abundant proteins (DAPs) in filets treated to microwave thawing (MT), while 13 DAPs were detected in MNPMT. Bioinformatics analysis of Gene Ontology (GO) enrichment, Kyoto Encyclopedia of Genes and Genomes (KEGG) pathway enrichment, protein–protein interaction (PPI), and subcellular localization prediction of differentially abundant proteins (DAPs) revealed that many DAPs were linked to protein structure, metabolic enzymes, and protein turnover, among other functions [106]. Organic-coated superparamagnetic iron oxide nanoparticles (OC-SPIONs) were produced and studied using transmission electron microscopy and X-ray photoelectron spectroscopy. OC-SPIONs were transitioned from organic media to aqueous solution utilizing poly(amidoamine) dendrimers modified with 6-TAMRA fluorescent dye and folic acid moieties [107]. The saturation magnetization of the dendrimer-coated SPIONs (DC-SPIONs) was measured using a superconducting quantum interference device, yielding a value of 60 emu/g Fe compared to 90 emu/g Fe for bulk magnetite (Figure 10). The selective targeting of DC-SPIONs to KB cancer cells in vitro was proven and quantified using two distinct imaging modalities: UV–visible and X-ray fluorescence, and confocal microscopy verified internalization.

**Figure 11:**
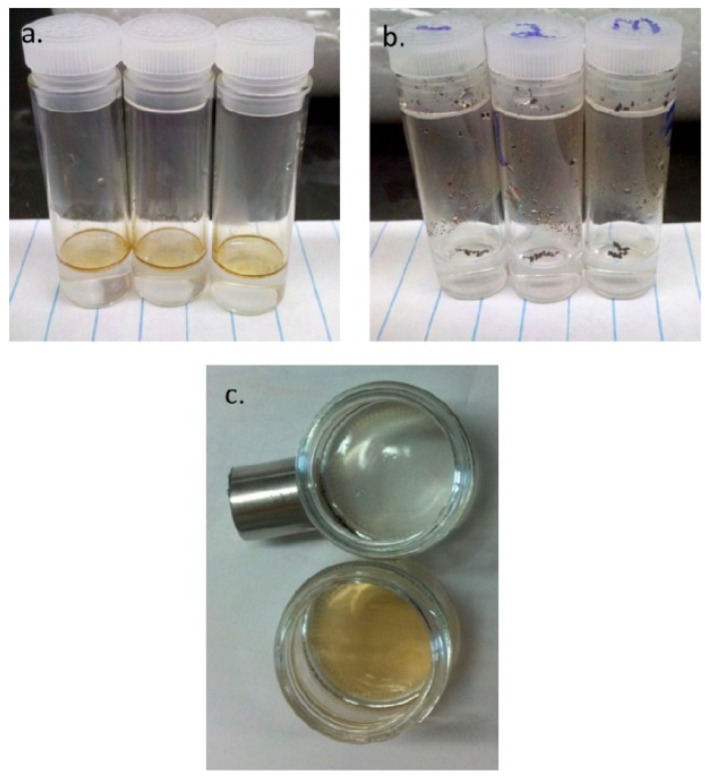
Images of oil sequestration experiment: (**a**) vial containing crude oil-contaminated water; (**b**) image showing vial after oil sorption by hybrid MSCK nanoparticles; (**c**) top view comparison of crude oil-loaded nanoparticles captured against the vial wall by an external magnet (**top**) and crude oil-contaminated water (**bottom**) [110].

The clinical classification of cardiac troponin I (cTnI) levels is crucial for the precise diagnosis of acute myocardial infarction. This study presents a novel point-of-care test that integrates an engineered locked aptamer, magnetic nanoparticles, and isothermal, non-enzymatic hybridization chain reaction with surface-enhanced resonance Raman scattering for the sensitive and specific detection of cTnI at clinically relevant concentrations. The high-sensitivity cTnI assay facilitated precise quantification of cTnI in 25 μL of cTnI-spiked serum, exhibiting a detection limit of 0.403 ng/L and a quantification limit of 1.22 ng/L, with a dynamic range extending from 0.5 ng/L to 50,000 ng/L. The assay, tailored to categorize cTnI levels exceeding the accepted clinical threshold of 40 ng/L, effectively differentiates between healthy and unhealthy clinical samples, attaining over 83% accuracy and 86% precision [108]. The manufacturing method of nanoparticles in solution via laser ablation of a bulk target material submerged in a liquid does not necessitate the use of reducible chemical precursors or colloidal stabilizers. The resultant colloids are ultrapure, and in specific instances, the produced nanoparticles possess surface ligand-free (or “bare”) surfaces. Moreover, laser irradiation of micro/nanomaterials dispersed in a liquid can result in the formation of nanoparticles in solution exhibiting distinctive physicochemical characteristics [109]. Interest in the application of the technology for producing magnetic nanoparticles generally stems from the following factors: Magnetic oxide or carbide nanoparticles can be directly produced using laser ablation of the respective elemental target material in a suitable solvent. Magnetoplasmonic bimetallic alloy nanoparticles (e.g., FeAu), including those formed from immiscible elements in solid and liquid states (e.g., FeAg, CoAg), can be synthesized by ablating nonalloyed bimetallic targets. (iii) In the presence of an external magnetic field, chains and strands of nanoparticles can be formed, exhibiting a high packing density of nanoparticles, which leads to strands with elevated aspect ratios and electrical conductivity owing to their “bare” surfaces. Magnetic nanostructures consisting of nanoparticles and nanomaterials with diverse morphologies or characteristics can be synthesized. Nanoparticles of intrinsically nonmagnetic oxides that display d0 ferromagnetism can be produced. The utilization of laser-synthesized magnetic nanoparticles in liquid for the production of polymer–nanoparticle composites (magnetic nanocomposites) eliminates the occurrence of chemical reaction byproducts within the nanocomposite. The nanoparticles establish direct chemical connections with the polymer matrix. Magnetic shell cross-linked knedel-like nanoparticles (MSCKs) with hydrodynamic diameters of approximately 70 nm were synthesized via the co-assembly of amphiphilic block copolymers PAA20-b-PS280 and oleic acid-stabilized magnetic iron oxide nanoparticles utilizing tetrahydrofuran, N,N-dimethylformamide, and water, culminating in a completely aqueous system (Figure 11). These hybrid nanomaterials were engineered to function as sequestering agents for hydrocarbons in crude oil, leveraging their amalgamation of amphiphilic organic domains for dispersibility in aqueous solutions and the capture of hydrophobic guest molecules, alongside inorganic core particles for magnetic responsiveness [110].

## 5. Toxicological Considerations of MNPs in Food Applications

Magnetic nanoparticles (MNPs), especially iron oxide-based nanostructures (such as Fe_3_O_4_ and γ-Fe_2_O_3_), have transformed food preservation, packaging, and nutrition delivery, owing to their superparamagnetism, biocompatibility, and functional flexibility [111]. Their applications encompass active packaging solutions that impede microbial proliferation, sensors for pollutants, and transporters for nutraceuticals [112,113,114]. Nonetheless, their diminutive size (<50 nm) facilitates cellular incorporation through endocytic mechanisms (e.g., clathrin- or caveolin-mediated uptake), thus eliciting considerable toxicological apprehensions [115]. The possible migration of micro- and nanoplastics from food contact materials into consumables requires a thorough evaluation of their health effects, particularly in light of studies indicating bioaccumulation in human organs, including the liver, placenta, and gastrointestinal system [116]. This section consolidates existing research on the mechanisms of MNP toxicity, contributing factors, and regulatory obstacles to guide safer design and application in food contexts. MNPs provoke the formation of reactive oxygen species (ROS), owing to their elevated surface-area-to-volume ratio, surpassing intrinsic antioxidant defenses (e.g., glutathione peroxidase) [117,118]. This oxidative cascade inflicts damage on lipids, proteins, and DNA, instigating inflammation through the activation of the NF-κB pathway and the release of pro-inflammatory cytokines (e.g., TNF-α, IL-6) [119]. For example, Fe_3_O_4_ nanoparticles at concentrations over 100 µg/mL markedly diminished the survival of Caco-2 intestinal cells by 40–60% via mitochondrial membrane depolarization [120]. Prolonged contact to micro/nanoplastics can induce DNA strand breakage and chromosomal abnormalities [121]. Polystyrene MNPs (PS-MNPs) sized 1–10 µm augmented micronucleus formation in human cells by three times, signifying clastogenic properties [122]. Likewise, iron oxide magnetic nanoparticles lacking sufficient surface coating triggered apoptosis in hepatocytes through caspase-3 activation, worsening liver failure in mouse models. MNPs serve as vectors for co-contaminants, such as heavy metals (e.g., Pb, Cd), antibiotics, and persistent organic pollutants (POPs), owing to their adsorptive surfaces [123]. Synergistic toxicity is evidenced in both aquatic and terrestrial models: PE-MNPs infused with polycyclic aromatic hydrocarbons (PAHs) increased zebrafish embryo mortality by 50% relative to PAHs alone [124].

## 6. Summary and Future Outlook

Magnetic nanoparticle (MNP)-based technologies signify a transformative advancement in food preservation, providing novel solutions to persistent issues in nano-packaging and nano-freezing. This review illustrates how MNPs improve packaging systems via antibacterial, gas-scavenging, and real-time sensing functionalities, while concurrently transforming cryopreservation by regulating ice nucleation and facilitating rapid nanowarming. The essential findings indicate that MNP-integrated active packaging diminishes microbiological contamination by more than 99% [125], prolongs shelf life by as much as 50% [126], and enhances food safety via intelligent spoiling monitoring. MNP-integrated active packaging has demonstrated the ability to reduce microbiological contamination by more than 99%, significantly improving the microbial safety of stored foods. This high antimicrobial efficacy helps maintain product quality and prolongs shelf life, making it a promising approach for commercial food preservation. In cryogenic applications, MNPs mitigate cellular damage in seafood, meats, and produce by inhibiting ice crystal formation, thereby retaining texture, nutrition, and flavor. Commercial implementation is already in progress, with technologies such as ethylene-scavenging MNP sheets and magnetic field-assisted freezers exhibiting scalability. Nonetheless, obstacles including regulatory inconsistency, possible nanoparticle migration, and consumer acceptance must be resolved to enable extensive adoption.

**Figure 12:**
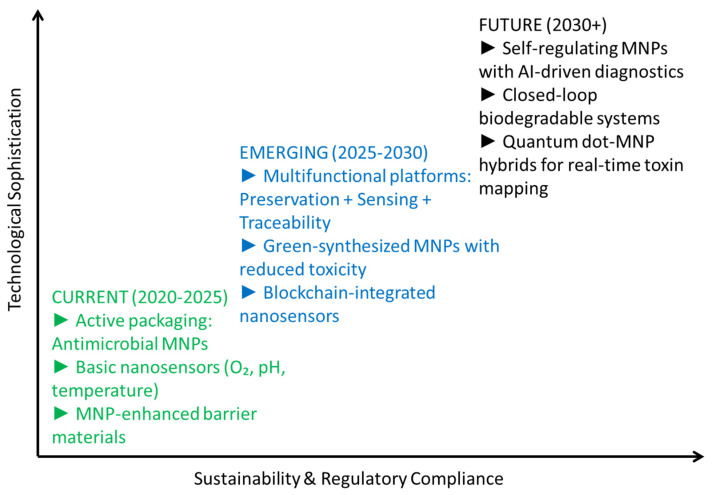
Evolution of MNP-enabled food preservation technologies.

The future of MNP-based food preservation depends on three essential advancements: (1) sustainable MNP synthesis through plant- and microbe-mediated production methods that decrease reliance on chemical processes and lessen environmental impact; (2) multifunctional hybrid systems, including MNP–biopolymer composites that integrate preservation, sensing, and biodegradability into one material; and (3) closed-loop recovery systems, wherein MNPs are magnetically extracted and reused from food packaging waste, in accordance with circular economy principles. Modified atmosphere packaging (MAP), often referred to as MNP, has undergone significant evolution since its commercial inception. Early forms in the mid-20th century relied primarily on vacuum sealing, simply removing air to slow oxidation and microbial growth (Figure 12). The true breakthrough came in the 1970s with the development of gas flushing techniques, allowing precise replacement of the internal atmosphere. Initially focused on simple gas mixtures like high carbon dioxide for meat or low oxygen for fresh produce, the technology rapidly advanced. Sophisticated machinery enabled tailored gas combinations (e.g., O_2_, CO_2_, N_2_) specific to the respiration rates and spoilage mechanisms of diverse products, from bakery goods to ready meals. Concurrently, material science delivered high-barrier films, like those incorporating EVOH, crucial for maintaining the carefully engineered gas composition over an extended shelf life. The late 20th and early 21st centuries saw further refinement: equilibrium MAP, where packaging permeability matched product respiration; active packaging components (scavengers, emitters) integrated within MNP; and the emergence of intelligent/smart MNP, incorporating sensors for freshness indicators or temperature monitoring. Today, evolution continues towards sustainable MNP solutions using biodegradable films, optimized gas mixtures for waste reduction, and smart labels enhancing traceability and safety, solidifying MNP’s role as a cornerstone technology in modern food preservation.

Migration testing for nanoparticles in food contact materials involves rigorous protocols. Initial characterization (size, distribution, and agglomeration) is followed by exposure of the material to food simulants (e.g., aqueous, acidic, fatty) under standardized time–temperature conditions mimicking its intended use. Migrated nanoparticles are then quantified using advanced techniques like ICP-MS, TEM, or SP-ICP-MS to detect ultra-low levels. Regulatory frameworks like those of the FDA (US) and EFSA (EU) generally require a demonstration that migration remains below specific thresholds to qualify for exemptions. A key limit is the 0.01 mg/kg (10 ppb) food migration threshold for substances requiring notification; nanoparticles often trigger stricter safety assessments due to their unique properties [127]. If migration exceeds thresholds, comprehensive toxicological data is mandated. Both agencies emphasize a case-by-case approach, requiring proof that migration does not pose a health risk at intended use levels.

Regulatory frameworks must advance to create global safety standards for MNP utilization in food contact materials, incorporating harmonized guidelines from the FDA, EFSA, and Codex Alimentarius. Moreover, consumer confidence can be cultivated through transparent labeling and public awareness initiatives that highlight the contribution of nanotechnology to minimizing food waste and enhancing sustainability. The subsequent research phase must emphasize cost-effective, large-scale MNP manufacturing to improve commercial feasibility, in conjunction with extensive toxicological investigations to guarantee safety. Innovations like self-healing nano-packaging, which mends microcracks during storage, and MNP-assisted cold chain monitoring, utilizing integrated nanosensors, could significantly enhance food logistics. Should these developments be actualized, MNP-enabled preservation may diminish worldwide food waste by 15–20%, thus bolstering global food security while preserving nutritional quality. The forthcoming decade will ascertain whether these technologies evolve from limited use to widespread adoption, a transformation reliant on interdisciplinary collaboration among scientists, industry leaders, legislators, and consumers. By tackling technical, legislative, and societal difficulties, MNPs can realize their potential as a fundamental component of next-generation sustainable food systems.

## Data Availability

No new data were created or analyzed in this study. Data sharing is not applicable to this article.
